# What Sanitation Has Done for Life?

**Published:** 1896-11

**Authors:** 


### What Sanitation has Done for Life.

For conciseness and force in dealing with the results of sanitation, the address of Prof. Brewer, of Yale University, recently delivered before the Life Association of Western Massachusetts, has rarely been excelled. The question has often been debated, but never satisfactorily answered, whether there was any substantial gain to human life from the ordinary medical methods of combating specific cases of disease, or whether the mere elimination of one form of disorder did not have its compensation in the development of some other. In respect to sanitation, however, there can be no dispute. The gain is unquestioned and immense. The span of life measured from infancy to old age may not have been lengthened, but the number of those dropping by the wayside has been so effectually reduced that life insurance rates to-day are not what they must have been a hundred years ago. We cull the following striking passages from the address of Prof. Brewer:

That human life has been prolonged by the application of science in the last fifty years, no one doubts. How this has come about forms an intensely interesting chapter in the history of our civilization. But it is not a simple story. How much, mathematically, this amounts to, in years, in per cents, is an unanswerable question. We can never have the data in figures. Even if we had our vital statistics completed for that period, men would differ as to the relative value of the several factors in this problem. Our great cities would not exist—they could not exist without the aids of science. As to what would take place, the answer is as uncertain as the answer to the question, "What would now be the condition of Europe if Napoleon had never been conquered?" "If Columbus had not discovered America, who would have discovered it?" What we can say is that science has wrought so many changes that our civilization of to-day is very much in advance of what it would have been without science, and the prolongation of human life is but one phase of the relations of science to modern civilizations. We have had an ancient Egyptian and Greek and Roman civilizations, which were pagan, and later Christian civilization, and all were powerless to convert practices. Between the epidemics that raged from time to time, and the high death rate in the best of years, the population of Europe, as a whole, probably scarcely increased at all for 1000 or 1200 years.

This century came in without a single city in Christendom with a million of inhabitants. Paris had, in 1800, but 548,009; London and its suburbs, in 1801, 864,845. The other great English cities had less than 100,000. Great cities could not endure then. First, the people could not be fed. Then, most of the population had to be fed and food produced within twenty miles of the place of consumption. Science has now made it possible to transport food half way around the globe, and has discovered new methods of preservation as well. City population was not self-perpetuating. Man died off; the death rate was continually high, and from time to time there was death by pestilence. Even where there were sewers, they were to drain the ground of water, rather than to carry away sewage. Now cities are made nearly as healthy as the country. Another reason why there could be no great cities in the modern sense related to business matters purely. I have dwelt upon this phase because cities are containing a larger and larger proportion of the inhabitants of civilized countries. And human life in cities is much prolonged. It is a law long ago demonstrated that the death rate increases as the density of population increases. This is still true, bnt sanitary science has enormously diminished this rate.

Sanitary science has been of slow growth but of rapid fruitage. It was 200 years ago that a Dutch scientist discovered the yeast scell, but the actual significance and working of the cell was not understood until 150 years later. In 1837 it was discovered that in the yeast cells were living organisms which were multiplying in the fluid in which they grew. This was the cause of fermentation. For years chemists quarreled as to whether these germs were the cause or the effect of this fermentation, but the former theory is now fully established. Then another dispute arose as to whether life could originate in decaying animal and vegetable matter, but it was finally proved that this did actually occur.

This theory forms the foundation of the modern disinfecting processes, and from this time diseases of all kinds were studied in a new light and given radically different treatment. In 1849 and 1850 French scientists showed that the blood of animals who had died of anthrax contained small particles, and ten years later it was discovered that these particles, when introduced into a living organism, would cause the disease. In 1870 it was proved that putrefaction, like fermentation, was due to germs. In the same year the germ of relapsing fever, one of the most dangerous diseases on the continent, was discovered, and during the decade following there was much further investigation along the same lines.

The germ for leprosy was discovered in 1879, for typhoid fever in 1880, for consumption in 1882, and for cholera in 1884.

This last discovery was the greatest discovery of all, and it resulted from the heroic efforts of scientists who went to Egypt to investigate the disease while it was raging there in 1883. But the accuracy of the theory was not generally accepted until 1800. Now we know the cause of the disease, and there can be no excuse hereafter for an epidemic of cholera in any of our cities. When there was cholera in Hamburg a few years ago, the commercial interests of England were of such vast importance that she refused to quarantine her ports against the disease. Hundreds of cases were brought into England, but the disease was so thoroughly understood that it was stamped out as soon as it appeared in every case, and there was no epidemic. To what extent human life, in the aggregate, has been prolonged by better food, and more of it, improvement in sanitation, and the advances made in the scientific treatment of disease, can never be statistically determined. But it is certain now that diseases are due to the operation of causes which are pretty well understood. Cities understand that they can no longer afford to have bad sanitation, and these improvements alone mean the prolongation of the working periods of men's lives.—*Insurance Monitor.*

